# The Ecuadorian Version of the Burnout Assessment Tool (BAT): Adaptation and Validation

**DOI:** 10.3390/ijerph18137121

**Published:** 2021-07-02

**Authors:** Andrea M. Vinueza-Solórzano, Cecilia Alexandra Portalanza-Chavarría, Clarissa P. P. de Freitas, Wilmar B. Schaufeli, Hans De Witte, Claudio S. Hutz, Ana Claudia Souza Vazquez

**Affiliations:** 1 Department of Psychology, Universidade Federal de Ciências da Saúde de Porto Alegre, Porto Alegre 90050-170, RS, Brazil; 2 Research Center, University Espiritu Santo, Samborondón 09-01-952, Ecuador; aportalanza@uees.edu.ec; 3 Department of Psychology, Pontifical Catholic University of Rio de Janeiro, Rio de Janeiro 22451-900, RJ, Brazil; freitas.cpp@gmail.com; 4 Research Group Work, Organizational and Personnel Psychology, FPPW, KU Leuven, 3000 Leuven, Belgium; wilmar.schaufeli@kuleuven.be (W.B.S.); hans.dewitte@kuleuven.be (H.D.W.); 5 Department of Social and Organizational Psychology, Utrecht University, 3584 CS Utrecht, The Netherlands; 6 Optentia Research Unit, North-West University, Vanderbijlpark 1900, South Africa; 7 Department of Psychology, Universidade Federal do Rio Grande do Sul, Porto Alegre 90035-002, RS, Brazil; claudio.hutz@gmail.com

**Keywords:** Burnout Assessment Tool, adaptation, factorial validity, reliability, construct validity

## Abstract

This study aimed to adapt and show evidence of validity for the Ecuadorian version of the Burnout Assessment Tool (BAT) considering only its “core” dimensions. The adaptation process included its translation and back translation. For content validation, expert reviews and focus groups were carried out. A confirmatory factor analysis was used to identify the psychometric properties and dimensionality of the scale. The reliability of the scale was assessed through the alpha, omega and composite reliability indices. To carry out the study, the questionnaire was applied to a sample of workers with a high level of education in Ecuador. In total, 2237 respondents were considered in the analysis. The results showed that the hierarchical model for BAT-23 and its short version, the BAT-12 scale, is the most adequate structure for analysis of the construct in the Ecuadorian context. The reliability of the general factor of burnout and its dimensions, evaluated by composite reliability, omega and Cronbach’s alpha, showed satisfactory indices. The findings obtained provide support for the reliability and validity of the Burnout Assessment Tool for the Ecuadorian context.

## 1. Introduction

In recent decades, the work overload of different professions and occupations has increased to achieve greater productivity for organizations [1]. However, this leads to contrary results, causing workers’ low performance and sometimes affecting their health [2]. When the demands of the environment increase and exceed a person’s ability to face their work, it can cause burnout and bring consequences such as rotation, resignations, non-compliance, and absences due to health problems and family problems [3]. The World Health Organization classifies burnout syndrome (BS) as a disease resulting from workplace stress that has not been correctly managed [4].

Burnout was described for the first time in the United States by Freudenberger in 1974 as “Staff Burnout” [5]. It consists of exhaustion and is caused by excessive demands that require greater energy use. Burnout occurs when professionals become stressed and burn a disproportionate amount of energy, which results in fatigue and can cause failure to meet work goals. However, two scientific fields have contributed to important advances: (1) studies on psychological stress reactivity and (2) investigations on the clinical validity of evaluations of burnout. Turner et al. (2020), in a systematic review of prospective evidence, showed that health and disease outcomes can be predicted by activation of the sympathetic–adrenal–medullary (SAM) axis and the hypothalamic–pituitary–adrenal (HPA) axis in dealing with stressors. As a type of chronic stress, burnout can be biologically explained by the hyper-responsiveness of the HPA axis, which needs a continuing stressor and is only activated in more extreme circumstances [6,7]. Earlier identification of burnout symptoms and its stressors was performed by Schaufeli, Desart and De Witte (2020) in their proposal of a comprehensive burnout diagnosis that “allows making a distinction between healthy employees and those who run a large risk of burning-out”. This requires clinically validated cut-off scores that can be calculated to discriminate “cases” from “non-cases” with specificity and sensitivity. Therefore, the Burnout Assessment Tool (BAT) was designed as a diagnostic instrument and, at the same time, a potential preventive screening tool [8].

Due to COVID-19, people around the world have developed new ways of completing work activities. Companies shifted their work environment abruptly from face-to-face work to remote work. These changes could affect the health and well-being of the workers [9]. In this sense, it is essential to evaluate employees’ health periodically and understand the burnout syndrome prevalence.

Although various instruments to recognize burnout have been developed in different countries, some have theoretical and practical problems, such as the Maslach Burnout Inventory (MBI), which has been systematically criticized for its conceptual, technical and psychometric shortcomings [8,10,11]. Additionally, Sharma et al. (2020) suggested a need for novel and innovative research approaches to explore burnout during a pandemic such as COVID-19, considering that it could impact all kinds of professionals’ mental health [11]. The development, validation and psychometric properties of new instruments will contribute to this research area [12,13]. In this line, Schaufeli et al. (2020) developed the Burnout Assessment Tool (BAT), a new self-report questionnaire to measure burnout based on a novel theory that overcomes these critiques on its assessment. Considering the BAT as a promising instrument in this field, this study aims to analyze its validity in the Ecuadorian context. 

Schaufeli, Desart and De Witte (2020) introduced a new definition using a dialectical approach and established four core dimensions (exhaustion, mental distance, cognitive impairment and emotional impairment) and three secondary dimensions (psychological distress, psychosomatic complaints and depressed mood), that constitute the BAT basis. This study will only focus on the former, as these are the core symptoms of burnout. However, it is worthy to point out that introducing a distinction between the core and secondary symptoms of burnout to the literature improves the accuracy of its evaluation and diagnosis. The authors define burnout as a “work-related state of exhaustion that occurs among employees, characterized by extreme tiredness, reduced ability to regulate cognitive and emotional processes, and mental distancing”. Its psychometric properties have been evaluated in countries such as Germany, Austria, Ireland, Finland, Japan, the Netherlands and Belgium [14]. Nevertheless, more studies should be considered to expand the analysis with countries in the Americas. In the framework of the Job Demands-Resources model (JDR), and the positive psychology perspective of “work” as a construct, the relationships between protective occupational variables and resources have high importance as some of the predictors of burnout [15,16]. This knowledge could help prevent burnout and protect workers’ well-being and health [17,18].

In Ecuador, the working population is exposed to various environmental and labor factors that deteriorate health and wellness, such as those related to violence, safety at work, employment conditions, political instability and social disorder in general. In this sense, the state’s role is to strengthen public health promotion policies and prevent diseases in the workplace [19,20,21]. 

Based on the considerations above, this study aimed to adapt and validate the Burnout Assessment Tool (BAT) in the Ecuadorian context. Due to the focus of this study—contributing to improve the accuracy of diagnosis of the main symptoms of burnout—we consider only its “core” dimensions of exhaustion, cognitive impairment, emotional impairment and mental distance. In addition, we examined the convergent validity of BAT-23 and BAT-12 by relating the scores of burnout and its dimensions to theoretically related constructs.

A positive relationship between burnout and its dimensions with quantitative demands and qualitative demands is expected. Job demands are conceptualized as job activities that require continual levels of physical, emotional or mental effort to ensure their development, which may cause different levels of physiological and psychological stress. Due the physiological and psychological costs, job demands of high levels are positively associated with the development of burnout. Examples of job demands are quantitative demands such as work overload, work underload and pace of change, and qualitative job demands related to the activities and job characteristics, such as mental, emotional and physical demands [15,16].

In addition, burnout and its dimensions are expected to be negatively associated with social resources and job content resources. The work resources defined as social resources refer to job clarity, team support, supervisor support, team spirit perception and teamwork, and the job content resources refer to job control and the perception about decision making. Work resources are characterized as aspects of the job that develop the role of a protective factor against the negative impacts of demands and contribute to professionals achieving their occupational goals, as well as seeking professional development and personal growth [15,16].

On the other hand, the relations of burnout and its dimensions with personal resources were explored through the association of this state with dispositional hope. Studies show that low-hope individuals are more susceptible to burnout, and that hope acts as a predictor of work engagement [22,23]. It was proposed in this study that burnout and its dimensions would show a negative relationship. Hope is characterized as a personal resource oriented to the future; it encompasses both thoughts directed at people’s goals and objectives as well as the individual’s set of beliefs about the possibility of their goals being achieved. Hope has the potential to promote a motivational state that helps individuals plan ways and develop actions that help them to achieve their goals [24]. Evidence shows that hope may act as a protective factor against the development of burnout [25,26].

Since burnout and work engagement are opposing work-related mental states, we investigated whether burnout and its dimensions were negatively associated to work engagement scores. Work engagement is a positive, fulfilling, affective–cognitive work-related state of mind [27]. Work engagement may be conceptualized as opposite to burnout because engaged employees tend to work hard (vigor), be highly involved (dedicated) and feel engrossed (absorbed) in their work. On the other hand, burned-out professionals may present extreme tiredness towards occupational activities (exhaustion), difficulties to regulate their cognitive process during work (cognitive impairment), a decrease in their ability to regulate emotional processes (emotional impairment) and a lack of interest and involvement in job activities (mental distance).

## 2. Method

### 2.1. Participants

A convenience sample of workers enrolled in a postgraduate part-time program in a private university in Guayaquil, Ecuador was analyzed. It was observed that from the total of 3644 people who opened and started the questionnaire, 2421 completely answered it, yielding a response rate of 66.44%. Finally, only 2237 of the participants met the inclusion criteria with a work status that required that they would be performing paid occupational activities autonomously or in an organization at the time of the research.

In the final sample, the mean age was 34 (SD = 6.8), 34.4% (*n* = 770) identified as men and 65.6% (*n* = 1467) identified as women. In relation to marital status, 48.6% (*n* = 952) were single, 42.0% were married (*n* = 940), 8.3% (*n* = 186) were divorced, 5.3% (*n* = 118) were in a common-in-law relationship, 1.1% (*n* = 25) were separated and 0.7% (*n* = 16) were widowed. Most of the participants in the sample (86.1%, *n* = 1925) had a postgraduate degree, 13.8% (*n* = 308) had a university degree and only 0.1% (*n* = 4) had a specialization or PhD degree. At the time of the survey, and for this study’s purposes, 2,237 participants with an active work status were considered, in which we identified 75.2% (*n* = 1683) under a full-time contract, 6.9% (*n* = 154) with a part-time contract, 10.1% (*n* = 226) working under a professional services contract and 7.8% (*n* = 174) working as autonomous workers.

### 2.2. Data Collection

The survey was conducted between September and October 2020. All participants answered the instruments through a web-based platform. Participation was voluntary and all participants were asked to indicate that they agree with the online Informed Consent Form, where they were informed about the importance and objectives of the research and its confidentiality nature. The survey took about 15 min to complete. The data collected had a validation stage to exclude those with non-valid information or with missing information.

### 2.3. Ethical Considerations

The Scientific Committee of Research and Publications from the University Espiritu Santo, Samborondón, Ecuador, approved the study of the project named “Factores de Bienestar Laboral en Ecuador”, code no. 2021-ECON-002 (20/04/2021). The respondents were invited to participate on a voluntary basis. The individuals who agreed to participate responded to the instrument after signing the online Informed Consent Form.

### 2.4. Measures and Instruments

The Burnout Assessment Tool [28] was developed to measure burnout as a general score and to assess each of its core dimensions (exhaustion, mental distance, cognitive impairment and emotional impairment) and its secondary dimensions (psychological distress, psychosomatic complaints and depressed mood). This study only focuses on the “core dimensions”. The BAT has a long version that consists of 23 items and a short version that has 12 items. BAT-23 assesses exhaustion evaluated by 8 items (items 1 to 8), mental distance evaluated by 5 items (items 9 to 13), cognitive impairment evaluated by 5 items (items 14 to 18) and emotional impairment evaluated by 5 items (items 19 to 23) [28]. In BAT-12, the exhaustion dimension covers 3 items (items 1 to 3, equivalent to items 1, 3 and 4 from BAT-23), mental distance covers 3 items (items 4 to 6, equivalent to items 5, 9 and 13 from BAT-23), emotional impairment covers 3 items (items 7 to 9, equivalent to items 14, 17 and 18 from BAT-23) and cognitive impairment covers 3 items (items 10 to 12, equivalent to items 19, 20 and 22 from BAT-23) [29]. Participants answer the items on a scale of 1 (never) to 5 (always). The original BAT study concluded that the internal consistency of BAT-12 is very good (α > 0.92) but somewhat lower, by definition [29], than the internal consistency of BAT-23 (α > 0.97) [7]. This is because α depends on the number of items of the scale; the fewer the items, the lower the value of α is.

Work engagement was assessed with the short version of the Utrecht Work Engagement Scale translated into Spanish and validated for the Ecuadorian context, consisting of nine items [30,31]. The items are answered on a seven-point Likert scale, ranging from 1 (never) to 7 (always). In the present study, the psychometric proprieties of the scale were adequate (CFI = 0.97, TLI = 0.96, SRMR = 0.057 and RMSEA (90% C. I.) = 0.214 (0.208–0.221)). The internal consistency index presented an α = 0.923, Ω = 0.924 and CR = 0.956.

Adaptation and validation studies were carried out in the Ecuadorian context for the Snyder (1991) Dispositional Hope Scale, which validated the unifactorial structure model tested on a theoretical assumption’s basis for the possibilities of future studies. This scale has 12 items answered on a five-point Likert-type scale, in which 1 means totally false and 5 indicates totally true. The internal consistency of the validated instrument was (Cronbach’s alpha) α = 0.92 (0.91–0.92). The scale showed satisfactory psychometric proprieties in this sample (α = 0.697, Ω = 0.703, CR = 0.909, CFI = 0.942, TLI = 0.919, SRMR = 0.046 and RMSEA (90% C. I.) = 0.126 (0.118–0.134)).

Demands at work were evaluated through work overload, work underload, pace of change and the qualitative emotional, mental and physical demands from the Job Demands-Resources Questionnaire (JDR-Q). The subscales summed a total of 10 items. Job resources were evaluated through the social resource subscale and the content resources subscale from the Job Demands-Resources Questionnaire (JDR-Q). The social resources subscale evaluated team support, supervisor support and the team spirit and teamwork perception through 14 items. The subscale control at work assessed professionals’ perceptions on decision making and role clarity through 8 items. All items were answered on a Likert scale from 1 (totally disagree) to 5 (totally agree) points [30]. In the present study, the psychometric proprieties of the scale were acceptable for the Ecuadorian context, showing fit indices as follows: CFI = 0.90, TLI = 0.89, SRMR = 0.104 and RMSEA (90% C. I.) = 0.104 (0.102–0.104). The demand subscale presented an α = 0.788, Ω = 0.803 and CR = 0.977. The social resources subscale showed an α = 0.764, Ω = 0.780 and CR = 0.977, and the content resources subscale showed α = 0.916, Ω = 0.940 and CR = 0.977.

### 2.5. Procedures

*Translation and Adaptation*. The original BAT-23 measuring the core burnout symptoms was translated by two certified English-to-Spanish translation experts. The first, second and fourth authors of this study synthesized the two translated versions into a preliminary adapted version. Subsequently, two bilingual specialists with experience in the field of psychological assessment and organizational psychology evaluated our synthesis with no changes suggested at this stage. Furthermore, the version translated to Ecuadorian Spanish was compared to the Spanish version of the BAT-12 (see Appendix A Table A1) [28] as a reference to the items already translated. 

To assess the content validity between the Spanish version of BAT-12 and the related items of the Ecuadorian version (see Appendix B Table A2), there was a semantic analysis session between the authors. As a result, no difference was identified in the translation of the items W_MD3, W_MD5, W_CC5 and W_EC1. The following items were kept as shown on the Spanish version: W_EX3, W_MD1, W_CC1 and W_CC4. On the W_EX4, W_EC2 and W_EC5 items, the specification of a work-related context was added. Finally, the Ecuadorian translation for the W_EX1 item was used.

A back-translated version of BAT-23 was sent to the authors of the scale. It was approved by the authors of the original instrument because it presented semantic and idiomatic equivalence to the original version of the scale. After obtaining the authors’ consent, the final version of the Ecuadorian Spanish version of BAT-23 was applied to a pilot group of professionals (*N* = 5) to investigate the content validity. The pilot group evaluated whether each item was clear and understandable and whether they were able to find a relationship with the associated symptom. Based on the pilot group’s suggestions, no significant changes were made, but some items were adjusted to more fully characterize the aspects related to the dimensions. 

*Data Analysis.* To assess the factorial validity and dimensionality of BAT-23 and BAT-12, a confirmatory factor analysis (CFA) was performed [32]. The estimation method used was the Weighted Least Squares Mean and Variance Adjusted (WLSMV) because it is sufficiently robust for ordinal data. In the present study, the fit indices of three models were evaluated for BAT-23 and BAT-12. The first model assessed the unifactorial structure of BAT-23 and BAT-12. In this model, all items constituted a general factor of burnout. The second model investigated had a second-order structure, with items having a loading on their expected theoretical dimensions, and the four factors loading onto a higher-order factor of burnout for BAT-23 and BAT-12. 

The goodness of fit of BAT-23 and BAT-12 was assessed using the following fit indices: Chi-squared/degrees of freedom (χ2/df) ratio, the comparative fit index (CFI), the Tucker–Lewis index (TLI) and the root mean square error of approximation (RMSEA). According to the guidelines used, the χ2/df value should be less than 3, the CFI and TLI values should be greater than 0.95 and the RMSEA value should be less than 0.08 to indicate acceptable fit (with a 90% confidence interval not greater than 0.10) [28].

To evaluate which model showed the best fit for BAT-23 and BAT-12, chi-squared difference test analyses (χ2) were conducted to verify if the goodness-of-fit indices of model 1 were significantly different from those of model 2 [33]. After confirming the best solution of BAT-23 and BAT-12, the reliability of the scale was assessed using ordinal Cronbach’s alpha (α), Omega (ϖ) and composite reliability (CR).

The evidence based on the relation with external variables was evaluated through convergent and discriminant validity. The convergent validity of BAT-23 and BAT-12 was assessed using the relations of the general score of burnout and its specific dimensions (exhaustion, mental distance, emotional impairment and cognitive impairment) with quantitative demands, qualitative demands, social resources, job content resources, work engagement and dispositional hope. It was expected that burnout and its specific dimensions would show positive relations of moderate magnitude with quantitative and qualitative demands. Additionally, burnout and its specific dimensions should present negative associations with social resources, job content resources, work engagement and dispositional hope. The correlations were investigated through two structural equation models to control the measurement error of the model, with one model for each version of the BAT. The evidence of discriminant validity of BAT-23 and BAT-12 was evaluated through comparison of the Average Variance Extracted (AVE) of each construct and the squared correlations (r^2^) of the constructs with each other. Evidence of discriminant validity is obtained when the values of AVE exceed the squared correlation between the variables [28]. All analyses described in this study were carried on R Studio version 4.0.2.

## 3. Results

### 3.1. Factorial Validity

The results of the first CFA that evaluated the unifactorial solution showed low goodness-of-fit indices for BAT-23 and BAT-12. The second model, which assessed a higher-order four-factor solution for BAT-23 and BAT-12 proposed by Schaufeli et al. (2019), presented adequate goodness-of-fit indices for BAT-23 and BAT-12 (Table 1).

The chi-squared difference test [27] evidenced that the goodness-of-fit indices of the second-order solution structure (model 2) of BAT-23 and BAT-12 were superior to those of the unifactorial solution (model 1) of BAT-23 and BAT-12. The chi-squared difference test results were statistically significant for the four comparisons (BAT-23, *χ*^2^(gl)M2xM1 = (4) 1609.1, *p* < 0.0001; BAT-12, *χ^2^*(gl)M2xM1 = (18) 510.43, *p* < 0.0001).

**Figure 1:**
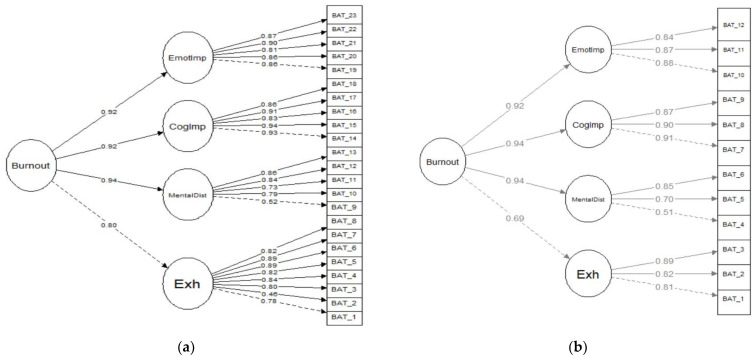
Burnout structural factor model for BAT-23 (**a**) and BAT-12 (**b**).

The second-order model assumes that the four distinct factors are indicators of one general, underlying factor (i.e., the core of burnout), which is supposed to be the cause of the correlation existing between the four factors. As shown in Figure 1a of the BAT-23 path diagram, the four dimensions loaded on the global burnout factor, with factorial loadings superior to 0.80. Analyzing the factorial loadings of the items, in the four factors, all items presented a factor loading higher than 0.45 (exhaustion, ranging from 0.46 to 0.89; mental distance, ranging from 0.52 to 0.86; emotional impairment, ranging from 0.81 to 0.90; and cognitive impairment, ranging from 0.83 to 0.94). The results in Table 1, Table 2 and Figure 1 show that BAT-23 and BAT-12 are adequate instruments to investigate burnout and its dimensions in the Ecuadorian context.

Furthermore, based on the goodness-of-fit indices and its brevity, it is suggested to use the short version of BAT-12 rather than the BAT-23 version. In BAT-12 (Figure 1b), all the dimensions showed a factor loading higher than 0.70 on global burnout. The factorial loading of the items in the specific dimensions of exhaustion (0.81 to 0.89), mental distance (0.51 to 0.85), emotional impairment (0.84 to 0.88) and cognitive impairment (0.87 to 0.91) were all higher than 0.50.

### 3.2. Reliability

The reliability of the scale was assessed using ordinal Cronbach’s alpha (α), Omega (ϖ) and composite reliability (CR) for the global factor of burnout and its dimensions assessed by BAT-23 and BAT-12. The convergent validity for BAT-23 and BAT-12 was evaluated separately. The results presented in Table 3 show the expected correlations between the dimensions and variables. 

### 3.3. Evidence Based on Relation with External Variables

The results showing the relationships between the external variables are demonstrated in Table 4 (p. 9). The level of burnout and its four specific dimensions as measured by BAT-23 and BAT-12 are positively associated with quantitative demands (work overload, work underload and pace of change) and qualitative job demands (mental, emotional and physical demands). Furthermore, level of burnout and its dimensions were also negatively associated with social resources (job clarity, team support, supervisor support, team spirit perception and teamwork) and job content resources (job control and the perception about decision making).

Burnout and its dimensions showed negative associations with work engagement and the dispositional hope variable, as expected. Interestingly, however, dispositional hope negatively correlated with burnout and positively correlated with work engagement. As expected, there is a higher impact on work engagement, showing evidence of its role as a positive factor to strengthen the motivational processes. 

## 4. Discussion

The present study aimed to adapt the Burnout Assessment Tool [27] to the Ecuadorian context and show evidence for the scale’s validity, dimensionality, reliability and relations with external variables. The items’ high factorial loadings, the dimensions’ reliability and the goodness-of-fit indices shown in the results corroborate that burnout is characterized as a syndrome constituted by its core dimensions (exhaustion, mental distance and emotional and cognitive impairment) [8,14]. All items of BAT-23 and BAT-12 showed a high factorial loading and adequate threshold variability. These results present evidence that the extended and brief versions of the BAT can evaluate a wide range of burnout states, as well as allowing for measuring the different levels of the dimensions of exhaustion, mental distance and emotional and cognitive impairment. 

The items’ high factorial loadings, the threshold variability and the goodness-of-fit indices provide support for the replication of the second-order burnout latent factor model based on the four latent components (exhaustion, mental distance and emotional and cognitive impairment) of the BAT-23 and BAT-12 tools in the Ecuadorian context. In addition, all the dimensions showed excellent alpha, omega and composite reliability indices, with the exception of the dimension of mental distance from BAT-12. These findings evidence that both versions of the BAT are reliable instruments to assess burnout and its dimensions [8,14].

Both versions of the BAT presented evidence of convergent validity with the external variables; as expected, burnout and its dimensions were significantly and positively associated with quantitative demands and qualitative demands [15,16]. On the other hand, as proposed, burnout and its dimensions were significantly and negatively related to social resources and job content resources [15,16], dispositional hope [25,26] and work engagement [27].

The negative impact of quantitative and qualitative demands related to the physiological and psychological costs of work demands was observed on the pattern of relationships between burnout and its dimensions. The findings evidence that quantitative and qualitative demands may contribute to professionals experiencing higher levels of exhaustion and, consequently, developing burnout syndrome [15,16].

The protective roles of social resources and job content resources were observed in their associations with burnout, exhaustion, mental distance, cognitive impairment and emotional impairment [15,16]. In view of Ecuador’s collectivist values, future studies should focus on comprehending differences in social resources and job content resources and on preventing burnout in the Ecuadorian context, evaluating the possibility that social resources have a greater impact when compared to other labor resources due to the importance of social support in Latin cultures [34].

The results also evidence that personal resources, such as dispositional hope, may act as an element preventing burnout [25,26]. Based on the relations of burnout and its dimensions with dispositional hope, it was understood that the potential of hope contributes to professionals establishing new ways of solving distressing situations and being motivated to act [24] as a personal resource that hinders the development of burnout syndrome.

The negative association of work engagement with burnout and its dimensions corroborates the understanding that burnout and work engagement encapsulate opposing work-related mental states. The moderate associations between engagement and burnout and its dimensions evidence that professionals experiencing burnout will lack the positive affective–cognitive work-related state of mind observed in engagement while going through a state of exhaustion, absence of interest toward work activities and difficulties in regulating their cognitive and emotional processes during work [27].

Evidence of the discriminant validity of BAT-23 and BAT-12 showed that the two versions of the instrument evaluated burnout and its dimensions as unique constructs [35]. The evidence indicated that the evaluation of burnout and its dimensions by both versions of the scale is theoretically and empirically related to external variables and, they may be identified as distinctive constructs.

The findings of the present study evidence that the Ecuadorian version of BAT-23 and BAT-12 constitutes a great tool to assess professionals’ levels of burnout, differentiating professionals with high- or low-level burnout scores and its dimensions [8,14]. The psychometric performance of BAT-12 in comparison to BAT-23 suggests that the brief version of the BAT may be the optimum tool to assess burnout as a global measure or for screening purposes, especially considering the goodness-of-fit indices of the second-order model of burnout and the discriminant and convergent analyses. The BAT-12 tool may also be preferable in evaluations that aim to cover several constructs. However, the low values of indices of internal consistency for the dimension of mental distance in BAT-12 may comprise a constraint in the use of the brief version of the BAT to assess the burnout dimension in the Ecuadorian context. Based on these findings, researchers assessing Ecuadorian professionals are encouraged to use BAT-12 as a burnout screening tool and BAT-23 whenever the focus is on the evaluation of burnout dimensions.

The strengths of the study include the robustness of the data analysis procedures, in that all analyses were performed with corrections for the characteristics of ordinal and non-scalar variables. Furthermore, the sample of the study included different occupational groups, so it was possible to increase the variability of the relations of professionals with their work.

Although the study was based on a diverse and large sample, the use of a non-representative sample constitutes a limitation of the study. The use of a convenience sampling technique increases the probability that individuals who experience lower levels of burnout are more likely to voluntarily collaborate on the research. Another consequence of the convenience sample was that all participants had at least a university degree. The lack of less-educated professionals hinders the possibility of evaluating their comprehension of the BAT. Future studies should include a broader and more representative sample to deal with possible sampling bias in analyses of the psychometric propriety of the Ecuadorian version of the BAT.

Another limitation of this study is that the data were collected at one point only. This type of data does not allow us to develop a causal analysis and make inferences about the contribution of job and personal resources in buffering the impact of demand on the development of burnout.

Future studies could contribute to the reliability of results obtained on the mental distance subscale, which could be associated with the understanding of the items, or as a cultural characteristic from the Ecuadorians. It is also important to investigate if the low indices of internal consistency of mental distance are associated with the high education profile of the sample. Further studies might help to reach a clearer understanding about which factors are impacting the factorial validation processes to define the better-fitted structure, based on the ongoing debate about BAT dimensionality. The load on a second-order factor that assesses burnout, as proposed by Schaufeli et al. (2019), is also presented in studies where burnout was modeled as hierarchical, in line with the conceptual definition as a syndrome [8,14], providing evidence of adequacy as in this study. Other recent studies [36] showed that the bifactor model fits the best to the data, indicating a strong general factor, which is consistent with the idea that burnout is a syndrome comprising a set of related symptoms referring to one underlying psychological condition. Finally, there are also studies that, through the use of Rasch analysis, have shown that the core-symptoms–dimensions of the BAT constitute a unidimensional scale [12].

## 5. Conclusions

Due to the negative impact of the development of burnout on professionals, their close relations and the organizations for which they work, preventing burnout must be a matter of importance to organizations and HR from both research and practical perspectives. Based on this, the present study made advancements in providing initial evidence of the applicability of the Ecuadorian version of BAT-23 and BAT-12. The BAT was demonstrated to be a promising instrument to overcome various flaws in the traditional assessment devices. This study confirmed that the practical applicability of BAT-Ecuador can be observed when applying it as a single score for establishing its prevalence or a cut-off to be used as a screening tool. The findings presented show that the BAT constitutes a viable alternative tool for the assessment of burnout and its dimensions in the framework of occupational well-being and health development.

## Figures and Tables

**Table 1:** Confirmatory factor analysis of BAT-23 and BAT-12.

		df	*χ* ^2^	*χ*^2^/df	CFI	TLI	SRMR	RMSEA
**1**	**Unifactorial** **BAT-23**	230	9169.01 *	39.87	0.889	0.878	0.090	0.132 [90%, 0.130–0.134]
**2**	**Unifactorial** **BAT-12**	230	9169.01 *	39.87	0.879	0.867	0.101	0.133 [90%, 0.128–0.139]
**3**	**Second-order CFA BAT-23**	226	3316.72 *	14.68	0.962	0.957	0.050	0.078 [90%, 0.076–0.081]
**4**	**Second-order CFA BAT-12**	50	554.04 *	11.08	0.986	0.981	0.037	0.067 [90%, 0.062–0.072]

**Note**: * = *p* < 0.001.

**Table 2:** Standardized parameters of items of BAT-23 and BAT-12.

Item	F. L.	Threshold				Item	F. L.	Threshold			
		*t*_1_	*t*_2_	*t*_3_	*t*_4_			*t*_1_	*t*_2_	*t*_3_	*t*_4_
1	0.780 *	−0.893	0.029	1.132	2.007	1	0.812 *	−0.893	0.029	1.132	2.007
2	0.463 *	−1.278	−0.493	0.336	1.051	2	0.823 *	−0.706	0.258	1.071	1.819
3	0.797 *	−0.706	0.258	1.071	1.819	3	0.889 *	−0.639	0.393	1.339	2.110
4	0.837 *	−0.639	0.393	1.339	2.110	4	0.508 *	−0.485	0.143	0.509	0.890
5	0.821 *	−0.368	0.601	1.361	2.061	5	0.700 *	0.265	1.002	1.662	2.120
6	0.887 *	−0.043	0.928	1.666	2.269	6	0.855 *	0.629	1.384	2.007	2.582
7	0.893 *	−0.116	0.992	1.733	2.473	7	0.909 *	0.377	1.390	2.201	3.002
8	0.825 *	−0.852	0.252	1.079	1.713	8	0.895 *	0.182	1.323	2.080	2.650
9	0.524 *	−0.485	0.143	0.509	0.890	9	0.872 *	0.117	1.488	2.227	2.784
10	0.791 *	0.236	1.083	1.680	2.255	10	0.880 *	0.372	1.420	1.909	2.450
11	0.731 *	0.265	1.002	1.662	2.120	11	0.874 *	0.495	1.429	1.915	2.387
12	0.836 *	0.562	1.301	1.930	2.450	12	0.836 *	0.423	1.420	2.142	2.690
13	0.855 *	0.629	1.384	2.007	2.582						
14	0.928 *	0.377	1.390	2.201	3.002						
15	0.936 *	0.391	1.461	2.189	2.843						
16	0.829 *	0.130	1.256	1.999	2.473						
17	0.907 *	0.182	1.323	2.080	2.650						
18	0.863 *	0.117	1.488	2.227	2.784						
19	0.863 *	0.372	1.420	1.909	2.450						
20	0.859 *	0.495	1.429	1.915	2.387						
21	0.813 *	−0.045	1.009	1.780	2.300						
22	0.895 *	0.586	1.442	2.051	2.650						
23	0.868 *	0.423	1.420	2.142	2.690						

**Note:** * = *p* < 0.001; F. L. = factorial loading; τ = threshold.

**Table 3:** Internal consistency analysis.

Reliability		α (95% C.I.)	Ω (95% C.I.)	CR
Global Burnout	BAT-23	0.94 (0.93–0.94)	0.94 (0.93–0.94)	0.94
	BAT-12	0.87 (0.86–0.88)	0.86 (0.85–0.88)	0.93
**Specific Dimensions**				
Exhaustion	BAT-23	0.90 (0.89–0.91)	0.90 (0.89–0.91)	0.93
	BAT-12	0.84 (0.83–0.85)	0.84 (0.82–0.85)	0.88
Mental Distance	BAT-23	0.71 (0.69–0.73)	0.71 (0.68–0.73)	0.87
	BAT-12	0.52 (0.48–0.56)	0.53 (0.49–0.56)	0.74
Cognitive Impairment	BAT-23	0.91 (0.89–0.92)	0.91 (0.90–0.92)	0.67
	BAT-12	0.86 (0.84–0.87)	0.86 (0.85–0.88)	0.55
Emotional Impairment	BAT-23	0.88 (0.86–0.89)	0.88 (0.86–0.89)	0.93
	BAT-12	0.82 (0.79–0.84)	0.88 (0.86–0.89)	0.90

**Table 4:** Relationships between burnout, work engagement, dispositional hope and the job demands-resources model.

Variables		*M*	*SD*	AVE	1	2	3	4	5	6	7	8	9	10
1.Burnout	B23	1.85	0.56	0.97		0.88 **	0.83 **	0.83 **	0.83 **	0.31 **	−0.48 **	−0.37 **	−0.55 **	−0.35 **
	B12	1.79	0.58	0.97		[0.88, 0.89]	[0.82, 0.84]	[0.82, 0.84]	[0.81, 0.84]	[0.27, 0.34]	[−0.51, −0.44]	[−0.41, −0.33]	[−0.61, −0.48]	[−0.39, −0.31]
2.Exhaustion	B23	2.30	0.71	0.63	0.88 **		0.62 **	0.58 **	0.57 **	0.36 **	−0.39 **	−0.37 **	−0.50 **	−0.25 **
	B12	2.33	0.87	0.71	[0.88, 0.89]		[0.60, 0.65]	[0.55, 0.60]	[0.55, 0.60]	[0.32, 0.39]	[−0.43, −0.36]	[−0.41, −0.34]	[−0.53, −0.47]	[−0.29, −0.21]
3.Mental Distance	B23	1.78	0.67	0.57	0.83 **	0.62 **		0.63 **	0.63 **	0.21 **	−0.41 **	−0.30 **	−0.48 **	−0.30 **
	B12	1.87	0.78	0.49	[0.82, 0.84]	[0.60, 0.65]		[0.61, 0.66]	[0.60, 0.65]	[0.17, 0.25]	[−0.44, −0.37]	[−0.34, −0.27]	[−0.51, −0.45]	[−0.34, −0.26]
4.Cognitive Impairment	B23	1.55	0.63	0.80	0.83 **	0.58 **	0.63 **		0.75 **	0.18 **	−0.42 **	−0.28 **	−0.51 **	−0.36 **
	B12	1.51	0.62	0.80	[0.82, 0.84]	[0.55, 0.60]	[0.61, 0.66]		[0.73, 0.77]	[0.14, 0.22]	[−0.46, −0.39]	[−0.32, −0.24]	[−0.54, −0.48]	[−0.40, −0.33]
5. Emotional Impairment	B23	1.51	0.62	0.74	0.83 **	0.57 **	0.63 **	0.75 **		0.20 **	−0.41 **	−0.25 **	−0.48 **	−0.33 **
	B12	1.44	0.63	0.75	[0.81, 0.84]	[0.55, 0.60]	[0.60, 0.65]	[0.73, 0.77]		[0.16, 0.24]	[−0.45, −0.38]	[−0.29, −0.22]	[−0.51, −0.45]	[−0.37, −0.30]
6. Demands		3.37	0.62	0.35	0.32 **	0.38 **	0.21 **	0.18 **	0.22 **		−0.06 **	−0.09 **	−0.05 **	−0.01
					[0.28, 0.36]	[0.34, 0.41]	[0.17, 0.25]	[0.14, 0.22]	[0.18, 0.26]		[−0.10, −0.02]	[−0.13, −0.05]	[−0.10, −0.01]	[−0.05, 0.03]
7. Social Resources		4.22	0.66	0.68	−0.48 **	−0.39 **	−0.41 **	−0.42 **	−0.41 **	−0.06 **		0.62 **	0.57 **	0.38 **
					[−0.51, −0.44]	[−0.43, −0.36]	[−0.44, −0.37]	[−0.46, −0.39]	[−0.45, −0.38]	[−0.10, −0.02]		[0.60, 0.65]	[0.54, 0.60]	[0.34, 0.41]
8. Content Resources		3.80	0.88	0.69	−0.37 **	−0.37 **	−0.30 **	−0.28 **	−0.25 **	−0.09 **	0.62 **		0.46 **	0.24 **
					[−0.41, −0.33]	[−0.41, −0.34]	[−0.34, −0.27]	[−0.32, −0.24]	[−0.29, −0.22]	[−0.13, −0.05]	[0.60, 0.65]		[0.42, 0.49]	[0.20, 0.28]
9. Work Engagement		5.70	1.04	0.70	−0.58 **	−0.50 **	−0.48 **	−0.51 **	−0.48 **	−0.05 **	0.57 **	0.46 **		0.47 **
					[−0.61, −0.55]	[−0.53, −0.47]	[−0.51, −0.45]	[−0.54, −0.48]	[−0.51, −0.45]	[−0.10, −0.01]	[0.54, 0.60]	[0.42, 0.49]		[0.44, 0.50]
10. Dispositional Hope		4.46	0.54	0.59	−0.35**	−0.25 **	−0.30 **	−0.36 **	−0.33 **	−0.01	0.38 **	0.24 **	0.47 **	
					[−0.39, −0.31]	[−0.29, −0.21]	[−0.34, −0.26]	[−0.40, −0.33]	[−0.37, −0.30]	[−0.05, 0.03]	[0.34, 0.41]	[0.20, 0.28]	[0.44, 0.50]	

## Data Availability

The data presented in this study are available on request from the corresponding author.

## Appendix A

### Puntuación [Scoring Categories]

**Table A1:** Spanish version of BAT-12.

Nunca (Never)	Raramente (Rarely)	Algunas veces (Sometimes)	A menudo (Often)			Siempre (Always)		
1	2	3	4			5		
**Agotamiento (Exhaustion)**				**1**	**2**	**3**	**4**	**5**
En mi trabajo, me siento agotado/a mentalmente. (At work, I feel mentally exhausted)				□	□	□	□	□
Al final del día de trabajo, me resulta difícil recuperar mi energía. (After a day at work, I find it hard to recover my energy)				□	□	□	□	□
Me siento físicamente agotado/a en mi trabajo. (At work, I feel physically exhausted)				□	□	□	□	□
**Distancia mental (Mental Distance)**								
Me esfuerzo por encontrar entusiasmo en mi trabajo. (I struggle to find any enthusiasm for my work)				□	□	□	□	□
Siento una fuerte aversión hacia mi trabajo. (I feel a strong aversion towards my job)				□	□	□	□	□
Soy cínico sobre lo que mi trabajo significa para los demás. (I am cynical about what my work means to others)				□	□	□	□	□
**Deterioro cognitivo (Cognitive Impairment)**								
Tengo problemas para mantenerme enfocado en mi trabajo. (At work, I have trouble staying focused)				□	□	□	□	□
Cuando estoy trabajando, tengo dificultades para concentrarme. (When I’m working, I have trouble concentrating)				□	□	□	□	□
Cometo errores en mi trabajo, porque tengo mi mente en otras cosas. (I make mistakes in my work because I have my mind on other things)				□	□	□	□	□
**Deterioro emocional (Emotional impairment)**								
En mi trabajo, me siento incapaz de controlar mis emociones. (At work, I feel unable to control my emotions)				□	□	□	□	□
No me reconozco en la forma que reacciono en el trabajo. (I do not recognize myself in the way I react emotionally at work)				□	□	□	□	□
Puedo reaccionar exageradamente sin querer. (At work I may overreact unintentionally)				□	□	□	□	□

## Appendix B

### Puntuación [Scoring Categories]

Las siguientes frases están relacionadas con su situación laboral y cómo experimenta esta situación. Indique con qué frecuencia cada frase se aplica a usted. (The following statements are related to your work situation and how you experience this situation. Please state how often each statement applies to you.)

**Table A2:** Ecuadorian version of BAT-23.

Nunca (Never)	Raramente (Rarely)	Algunas veces (Sometimes)	A menudo (Often)	Siempre (Always)				
1	2	3	4	5				
**Síntomas centrales (Core Symptoms)**				**1**	**2**	**3**	**4**	**5**
**Agotamiento (Exhaustion)**								
En el trabajo me siento mentalmente exhausto. (At work, I feel mentally exhausted)				□	□	□	□	□
Todo lo que hago en el trabajo requiere de mucho esfuerzo. (Everything I do at work requires a great deal of effort)				□	□	□	□	□
Al final del día de trabajo, me resulta difícil recuperar mi energía. (After a day at work, I find it hard to recover my energy)				□	□	□	□	□
En el trabajo, me siento físicamente exhausto. (At work, I feel physically exhausted)				□	□	□	□	□
Cuando me levanto en la mañana, me falta energía para comenzar el día en el trabajo. (When I get up in the morning, I lack the energy to start a new day at work)				□	□	□	□	□
Quiero estar activo en el trabajo, pero por alguna razón no estoy en capacidad de controlarlo. [I want to be active at work, but somehow, I am unable to manage]				□	□	□	□	□
Cuando realizo mi trabajo, me canso más rápido de lo normal. (When I exert myself at work, I get tired quicker than normal)				□	□	□	□	□
Al final de mi jornada laboral, me siento mentalmente exhausto y agotado. (At the end of my working day, I feel mentally exhausted and drained)				□	□	□	□	□
**Distancia mental (Mental Distance)**								
Me esfuerzo por encontrar entusiasmo en mi trabajo. (I struggle to find any enthusiasm for my work)				□	□	□	□	□
En el trabajo, no pienso en lo que estoy haciendo y funciono en piloto automático. (At work, I do not think what I am doing and I function on autopilot)				□	□	□	□	□
Siento una fuerte aversión hacia mi trabajo. (I feel a strong aversion towards my job)				□	□	□	□	□
Me siento indiferente sobre mi trabajo. (I feel indifferent about my job)				□	□	□	□	□
Soy cínico sobre lo que mi trabajo significa para los demás. (I am cynical about what my work means to others)				□	□	□	□	□
**Deterioro cognitivo (Cognitive Impairment)**								
Tengo problemas para mantenerme enfocado en mi trabajo. (At work, I have trouble staying focused)				□	□	□	□	□
En el trabajo, me cuesta pensar con claridad. (At work I struggle to think clearly)				□	□	□	□	□
Soy olvidadizo y distraído en el trabajo. (I am forgetful and distracted at Work)				□	□	□	□	□
Cuando estoy trabajando, tengo dificultades para concentrarme. (When I’m working, I have trouble concentrating)				□	□	□	□	□
Cometo errores en mi trabajo porque tengo mi mente en otras cosas. (I make mistakes in my work because I have my mind on other things)				□	□	□	□	□
**Deterioro emocional (Emotional Impairment)**								
En el trabajo, me siento incapaz de controlar mis emociones. (At work, I feel unable to control my emotions)				□	□	□	□	□
No me reconozco en la forma que reacciono emocionalmente en el trabajo. (I do not recognize myself in the way I react emotionally at work)				□	□	□	□	□
Durante mi trabajo me pongo irritable cuando las cosas no salen como quiero. (During my work I become irritable when things do not go the way I want]				□	□	□	□	□
Me pongo molesto y triste en el trabajo sin saber por qué. (I get upset and sad at work without knowing why)				□	□	□	□	□
En el trabajo, puedo reaccionar exageradamente sin querer. (At work I may overreact unintentionally)				□	□	□	□	□
